# Development of a 3 RNA Binding Protein Signature for Predicting Prognosis and Treatment Response for Glioblastoma Multiforme

**DOI:** 10.3389/fgene.2021.768930

**Published:** 2021-10-18

**Authors:** Ruohan Sun, Yujun Pan, Long Mu, Yaguang Ma, Hong Shen, Yu Long

**Affiliations:** ^1^ Department of Neurology, the First Affiliated Hospital of Harbin Medical University, Harbin, China; ^2^ Department of Neurosurgery, the First Affiliated Hospital of Harbin Medical University, Harbin, China

**Keywords:** glioblastoma multiforme, RNA binding proteins, overall survival, risk score model, prognostic model

## Abstract

**Purpose:** Glioblastoma multiforme (GBM) is the most widely occurring brain malignancy. It is modulated by a variety of genes, and patients with GBM have a low survival ratio and an unsatisfactory treatment effect. The irregular regulation of RNA binding proteins (RBPs) is implicated in several malignant neoplasms and reported to exhibit an association with the occurrence and development of carcinoma. Thus, it is necessary to build a stable, multi-RBPs signature-originated model for GBM prognosis and treatment response prediction.

**Methods:** Differentially expressed RBPs (DERBPs) were screened out based on the RBPs data of GBM and normal brain tissues from The Cancer Genome Atlas (TCGA) and Genotype-Tissue Expression Program (GTEx) datasets. Gene Ontology and Kyoto Encyclopedia of Genes and Genomes analyses on DERBPs were performed, followed by an analysis of the Protein-Protein Interaction network. Survival analysis of the DERBPs was conducted by univariate and multivariate Cox regression. Then, a risk score model was created on the basis of the gene signatures in various survival-associated RBPs, and its prognostic and predictive values were evaluated through Kaplan-Meier analysis and log-rank test. A nomogram on the basis of the hub RBPs signature was applied to estimate GBM patients’ survival rates. Moreover, western blot was for the detection of the proteins.

**Results:** BICC1, GNL3L, and KHDRBS2 were considered as prognosis-associated hub RBPs and then were applied in the construction of a prognostic model. Poor survival results appeared in GBM patients with a high-risk score. The area under the time-dependent ROC curve of the prognostic model was 0.723 in TCGA and 0.707 in Chinese Glioma Genome Atlas (CGGA) cohorts, indicating a good prognostic model. What was more, the survival duration of the high-risk group receiving radiotherapy or temozolomide chemotherapy was shorter than that of the low-risk group. The nomogram showed a great discriminating capacity for GBM, and western blot experiments demonstrated that the proteins of these 3 RBPs had different expressions in GBM cells.

**Conclusion:** The identified 3 hub RBPs-derived risk score is effective in the prediction of GBM prognosis and treatment response, and benefits to the treatment of GBM patients.

## Introduction

As the most widespread malignant neoplasm in human brain, glioblastoma multiforme (GBM) has retained a severe incidence ratio and prognosis (Johnson et al., 2013; Davis, 2016). Even though therapeutic methods to GBM diagnosis and treatment are continually ameliorating, which includes surgical resection, temozolomide (TMZ) chemotherapy and radiotherapy, average survival of 15 months remains unsatisfactory (Kim et al., 2018; Ramos et al., 2018). Presently, diagnosis of GBM mainly relies on examination of histopathology, neoplasm molecular biomarkers and imaging assessments, which is certainly not applicable to early diagnosis (Batash et al., 2017). Thus, to improve the treatment effects and life quality of patients, more knowledge of GBM molecular mechanism is needed to create efficient approaches for early detection.

RNA binding proteins (RBPs) refer to the proteins that interact with several RNAs, and participate in most post-transcriptional modulation processes and cellular homeostasis (Hentze et al., 2018; Gebauer et al., 2020). RBPs mediate the modulation of RNA splicing, polyadenylation, stability, localization, translation, and degradation *via* binding to targeted RNAs and then forming ribonucleoprotein complexes (Wende et al., 2019). Taking post-transcriptional modulation into consideration, no doubt abnormally dysregulated RBPs are intimately associated with the incidence and development of many diseases, such as cancers (Pereira et al., 2017; Neelamraju et al., 2018). Recently, some researchers uncover that RBPs facilitate tumorigenesis not only by raising oncogene levels, but also by reducing tumor suppressor gene levels (Guo and Jia, 2018; Zhang et al., 2018). Therefore, a lot of attention has turned to the roles of RBPs in cancers.

It is reported that RBPs present a close relationship to glioma’s occurrence and development (Xu et al., 2018; Yi et al., 2018). For example, the expression of PCBP2 is dramatically increased at a higher stage of glioma. Ablating PCBP2 greatly decreases the colony formation and invasion capability of GBM cells (Han et al., 2013; Luo and Zhuang, 2017). HuR is overexpressed in high-grade malignancies (GBM and medulloblastoma). Additionally, HuR could bind and stabilize mRNAs of growth factors that are associated with the progression of brain neoplasm (Nabors et al., 2001). In glioma and medulloblastoma, MSI1 expression is significantly elevated (Kanemura et al., 2001). Based on these findings, we attempt to systematically investigate RBPs’ functions to understand their roles in GBM.

In current years, data mining and bioinformatics analysis have been largely applied in research on carcinomas. A large number of high-throughput data produced by microarrays and next-generation sequencing are gathered in public datasets. Among them, The Cancer Genome Atlas (TCGA) and Chinese Glioma Genome Atlas (CGGA) get extensively adopted. Exploration of tumor expression features and identification of prognostic indicators and biomarkers can be greatly aided by mining these data. Based on the methylation array data in TCGA dataset, Rajendra P. Pangeni et al. analyzed tumor subtype-associated epigenetic regulation in GBM bulk tumors using genome-wide methylation and transcription (Pangeni et al., 2018). Based on the Gene Expression Omnibus (GEO) database, Huiwen Gui et al. used bioinformatics analysis to determine that GAPDH, RHOA, RPS29, and RSS27A are the hub genes of Alzheimer’s disease (Gui et al., 2021).

Here, our study is to carry out a comprehensive analysis of GBM in TCGA and CGGA to determine the survival-related differentially expressed RBPs (DERBPs), and used a series of bioinformatics analyses, including Gene Ontology (GO), Kyoto Encyclopedia of Genes and Genomes (KEGG) and Protein-Protein Interaction (PPI), to get the hub RBPs and key pathways in GBM. Based on the selected hub RBPs and public data, we establish a risk model for prognosis and treatment response prediction.

## Materials and Methods

### Data Acquirement

We obtained 1,092 normal brain tissue samples of 201 individuals from the Genotype-Tissue Expression Program (GTEx, https://www.gtexportal.org/home/datasets), 153 GBM samples with relevant clinical data from TCGA (https://www.cancer.gov/about-nci/organization/ccg/research/structural-genomics/tcga), and 85 GBM samples with relevant clinical data from CGGA cohort (https://www.cgga.org.cn/download.jsp). 1542 RBPs were extracted from the above-mentioned 3 RNA-sequencing data cohorts (Li et al., 2020a). DERBPs were determined by the negative binomial distribution method between a normal brain and GBM tissues. Besides, the Limma package (http://www.bioconductor.org/packages/release/bioc/html/limma.html) was carried out here on the basis of the negative binomial distribution. It fits a universal linear gene model and utilizes empirical Bayes shrinkage for the assessments of interspersion and fold change (FC). We preprocessed original data by Limma package and ruled out genes with a mean value <1. Additionally, the DERBPs got determined by the Limma package with the standard criteria of |log_2_ FC| ≥ 1 and false discovery rate (FDR) < 0.05.

### KEGG Pathway and GO Enrichment Analyses

GO and KEGG got employed to evaluate the DERBPs’ biological functions. CC (cellular component), MF (molecular function) and BP (biological process) were the main classifications of GO. The clusterProfiler package in R participated in all enrichment analyses (Yu et al., 2012). Statistical significance was defined as *p* and FDR values <0.05.

### Construction of Protein-Protein Interaction Network

The DERBPs were input to the STRING database (Search Tool for the Retrieval of Interaction Gene, http://www.string-db.org/) (Li et al., 2020a) to determine PPI data. The construction and visualization of PPI networks were performed by Cytoscape 3.7.0 software.

### Prognostic Model Construction

To assess whether the DERBPs had an association with survival, univariate Cox proportional hazards regression analysis was carried out in TCGA GBM cohort for overall survival (OS). Then, the hub RBPs related to survival were further identified using multivariate Cox proportional hazards regression among candidate genes.

According to the gene signatures of survival-related hub RBPs determined by multivariate Cox proportional hazards regression, we established a risk score model as the formula described below.

$$\text{Risk score}=\overset{n}{\underset{i=1}{\sum }}\beta_{i}*E_{i}$$

“n” represents key RBPs number in total; “ $\beta_{i}$ ” indicates gene i ’s regression coefficient; “ $E_{i}$ ” represents the gene i ’s expression value. In the validation CGGA dataset, “ $\beta_{i}$ ” used was the same as that in the TCGA.

For exploring the prognosis and prediction abilities of risk score, we classified GBM patients into high- and low-risk groups on account of the median risk score survival analysis. Kaplan-Meier (KM) method with the log-rank test was conducted for the survival rate analyses of the two groups. Clinicopathological parameters and risk scores were analyzed through univariate and multivariate Cox regressions to ensure that risk scores possessed significance in clinical. In addition, the SurvivalROC (receiver operating characteristic) package completed the ROC curve analysis to evaluate the above model’s prognostic ability (Heagerty et al., 2000). A validation cohort of 85 GBM patients with responsible prognosis from CGGA was employed to confirm the prognostic model’s predictive capability. Lastly, RMS R package was utilized to generate the nomogram including calibration plots to predict OS.

### GBM Tissue Samples

The resected neoplasm specimens of two GBM patients were obtained from the Department of Neurosurgery, the First Affiliated Hospital of Harbin Medical University. Histological grading got classified according to WHO criteria. This experiment got the permission of the Institutional Review Board of Harbin Medical University, and written informed consent was signed by every patient. GBM-1 and GBM-2 cells were derived from patient fresh GBM specimens.

### Human Cell Lines

HEB (normal human glial cell) and U-87 MG (GBM cell) cells were kindly provided by Prof. Qian He (Shenzhen People’s Hospital, Shenzhen, China), all GBM cells were cultivated in DMEM with 10% FBS, penicillin (100 U/ml) and streptomycin (100 mg/ml) in a 37°C environment containing 5% CO_2._

### Western Blot

Tissue and cellular proteins were extracted using tissue extraction buffer and RIPA buffer with protease inhibitor cocktail (Sigma, P8340), respectively. SDS-PAGE was used to separate equal quantities of protein which was then transferred to nitrocellulose membranes. Antibodies applied here were listed as follows: anti-BICC1 (Sigma, HPA045212), anti-GNL3L (Sigma, SAB4502257), anti-KHDRBS2 (ThermoFisher Scientific, PA5-96508), anti-β-Actin (Sigma, A1978) and horseradish peroxidase-conjugated anti-rabbit/mouse IgG (Cell Signaling, 7074 and 7076). Compared with β-Actin, the expressions of target proteins were calculated and then generalized to the equivalent expressions in HEB cells.

### Statistical Analysis

The representative data were derived from five separate experiments and shown as the mean ± SEM. Graphpad Prism 7.0 or R software (https://www.r-project.org/) was adopted for statistical analysis. Unpaired Student’s t-tests with Mann-Whitney U tests and one-way ANOVA with Kruskal-Wallis H tests were used to examine differences between two or more groups. The correlation of risk score with clinicopathological parameters was studied by Pearson Chi-Square test and Fisher’s exact test. The log-rank test was for the statistical significance evaluation of the differences in each dataset, and the KM technique was used to create survival curves for the subgroups in each dataset. *p* < 0.05 indicated significance in statistics.

## Results

### Analysis of the DERBPs in Patients With GBM

**FIGURE 1:**
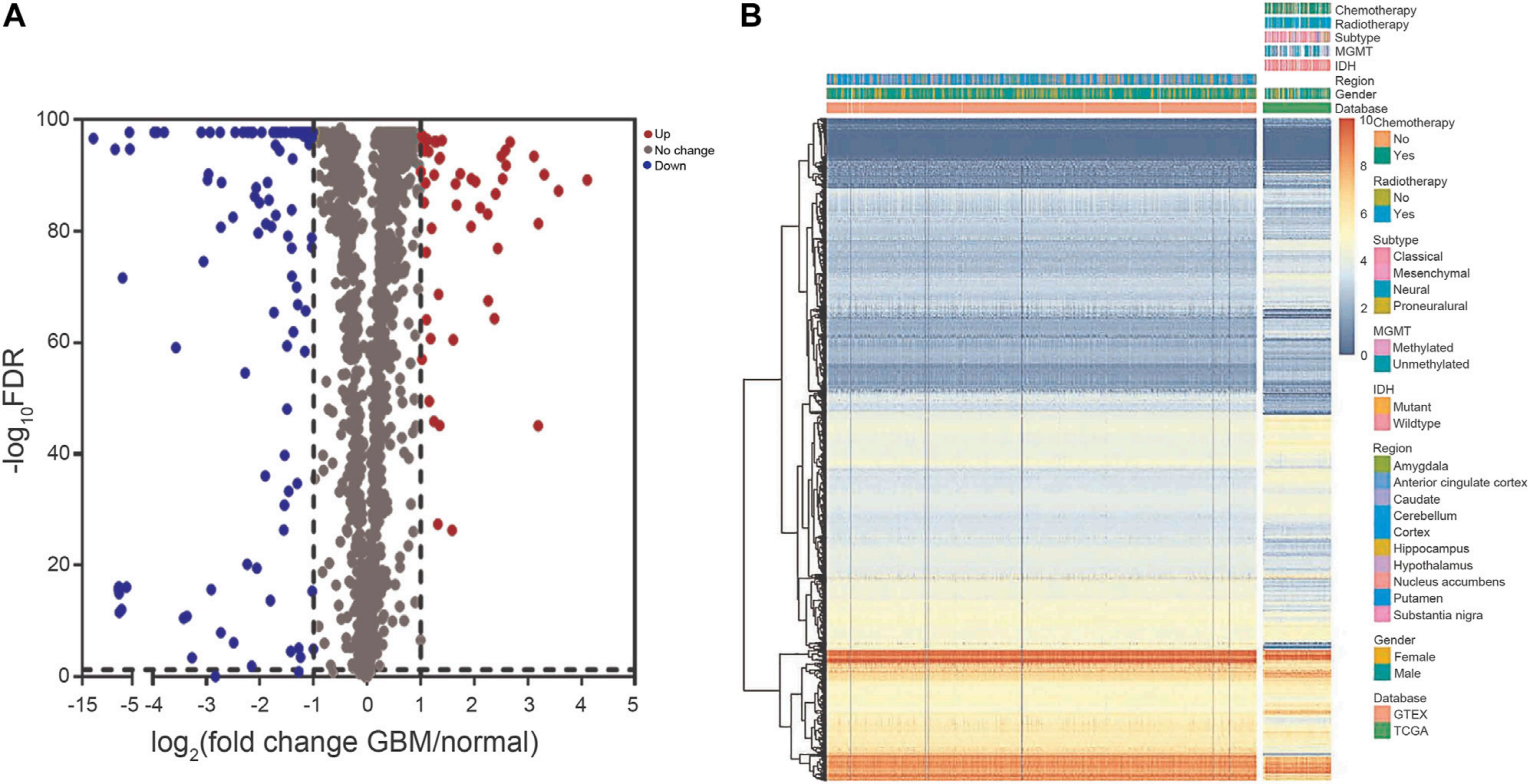
The DERBPs between GBM and normal brain tissues. **(A)** Volcano plot showing the log_2_FC of RBPs in GBM compared to normal brain tissues, and the corresponding–log_10_FDR in TCGA and GTEx datasets. Genes with FDR below 0.05 and log_2_FC above 1 (below -1) were marked with red (blue) dots. **(B)** Heat map of the RBPs in TCGA and GTEx datasets. DERBPs, differentially expressed RBPs; FC, fold change; FDR, false discovery rate.

The crucial prognosis role of RBPs in GBM was comprehensively studied by several advanced computational methods. Supplementary Figure S1 showed our study design. The GBM dataset (TCGA) contained 153 tumor samples in comparison to 1,092 normal brain samples (GTEx Portal). All derived data from the two datasets were preprocessed by the R software packages. In total, 1542 RBPs were analyzed and 160 RBPs were retained, consisting of 52 RBPs with upregulation and 108 RBPs with downregulation (Figure 1). Our data indicated GBM existed various DERBPs in comparison with normal brain tissues.

### Functional Enrichment Analysis of the DERBPs

**FIGURE 2:**
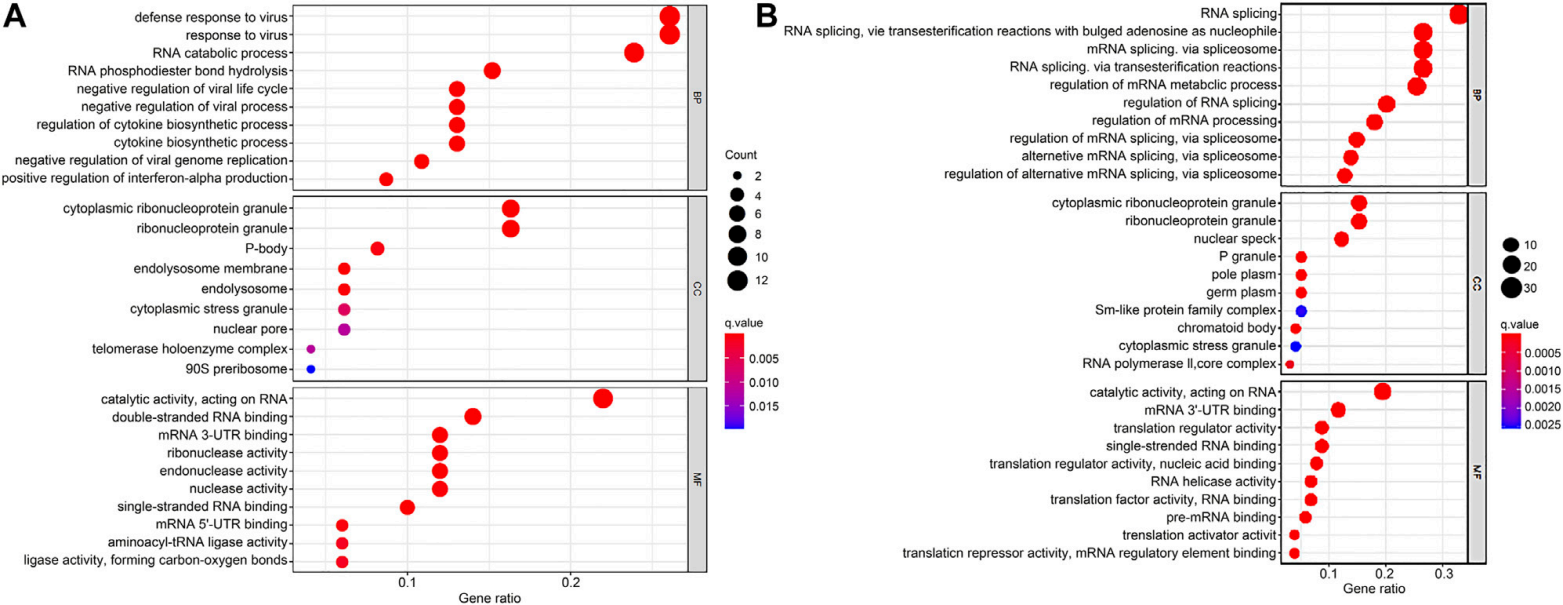
GO enrichment analysis of DERBPs based on BP, CC, and MF biological processes. **(A)** GO enrichment analysis of upregulated RBPs. **(B)** GO enrichment analysis of downregulated RBPs. The *y*-axis shows a significantly enriched project (*p* < 0.05, FDR < 0.05). GO, Gene Ontology; DERBPs, differentially expressed RBPs; BP, biological process; CC, cellular component; MF, molecular function.

The DERBPs comprised two groups of upregulation and downregulation. We conducted functional enrichment analysis of the two groups by GO and KEGG. Regarding GO analysis, BP, CC, and MF terms were applied to annotate these DERBPs’ functions. BP analysis demonstrated DERBPs with upregulation were primarily associated with defense response to viruses, RNA catabolic processes and RNA phosphodiester bond hydrolysis (Figure 2A), and that the DERBPs with downregulation were mainly related to the regulation of RNA splicing, RNA splicing and mRNA metabolic process (Figure 2B). By the CC analysis, both upregulated and downregulated DERBPs were enriched in total ribonucleoprotein granules and cytoplasmic ribonucleoprotein granules (Figure 2). MF analysis results showed DERBPs with upregulation were associated with catalytic activity acting on RNA, double-stranded RNA binding and mRNA 3′-UTR binding (Figure 2A), while those with downregulation were largely linked with catalytic activity acting on RNA, mRNA 3′-UTR binding and translation regulator activity (Figure 2B). The KEGG analysis demonstrated DERBPs with upregulation were significantly related to influenza A, mRNA surveillance pathway, ribosome biogenesis in eukaryotes and RNA transport. And those with downregulation were significantly associated with RNA transport, mRNA surveillance pathway, and RNA polymerase degradation (Table 1).

**TABLE 1:** KEGG pathway analysis of DERBPs.

Category	Term	Description	*p* Value	FDR	Count
Upregulated					
Pathway	hsa05164	Influenza A	2.86221E-06	7.53213E-05	7
Pathway	hsa03015	mRNA surveillance pathway	0.000382507	0.00503299	4
Pathway	hsa03008	Ribosome biogenesis in eukaryotes	0.000840096	0.007369263	4
Pathway	hsa05162	Measles	0.001824335	0.010607124	4
Pathway	hsa00970	Aminoacyl-tRNA biosynthesis	0.002015354	0.010607124	3
Pathway	hsa03013	RNA transport	0.00476635	0.020905044	4
Pathway	hsa04620	Toll-like receptor signaling pathway	0.007266019	0.02731586	3
Pathway	hsa05160	Hepatitis C	0.021283853	0.070012673	3
Pathway	hsa03018	RNA degradation	0.03688456	0.107849589	2
Downregulated					
Pathway	hsa03013	RNA transport	5.02482E-10	1.95703E-08	10
Pathway	hsa03015	mRNA surveillance pathway	8.49591E-07	1.65447E-05	6
Pathway	hsa03020	RNA polymerase	0.000196405	0.002549819	3
Pathway	hsa03018	RNA degradation	0.003062616	0.029820204	3
Pathway	hsa03008	Ribosome biogenesis in eukaryotes	0.008126664	0.063302434	3
Pathway	hsa03040	Spliceosome	0.017563625	0.10064299	3
Pathway	hsa05134	Legionellosis	0.018917424	0.10064299	2
Pathway	hsa05164	Influenza A	0.024818692	0.10064299	3
Pathway	hsa00970	Aminoacyl-tRNA biosynthesis	0.024904555	0.10064299	2
Pathway	hsa05016	Huntington disease	0.025840768	0.10064299	4

Note: Category: KEGG pathway. Count: the number of DERBPs.

Abbreviations: DERBPs, differentially expressed RNA binding proteins; KEGG, Kyoto Encyclopedia of Genes and Genomes.

### Selection of RBPs Related to Prognosis

**FIGURE 3:**
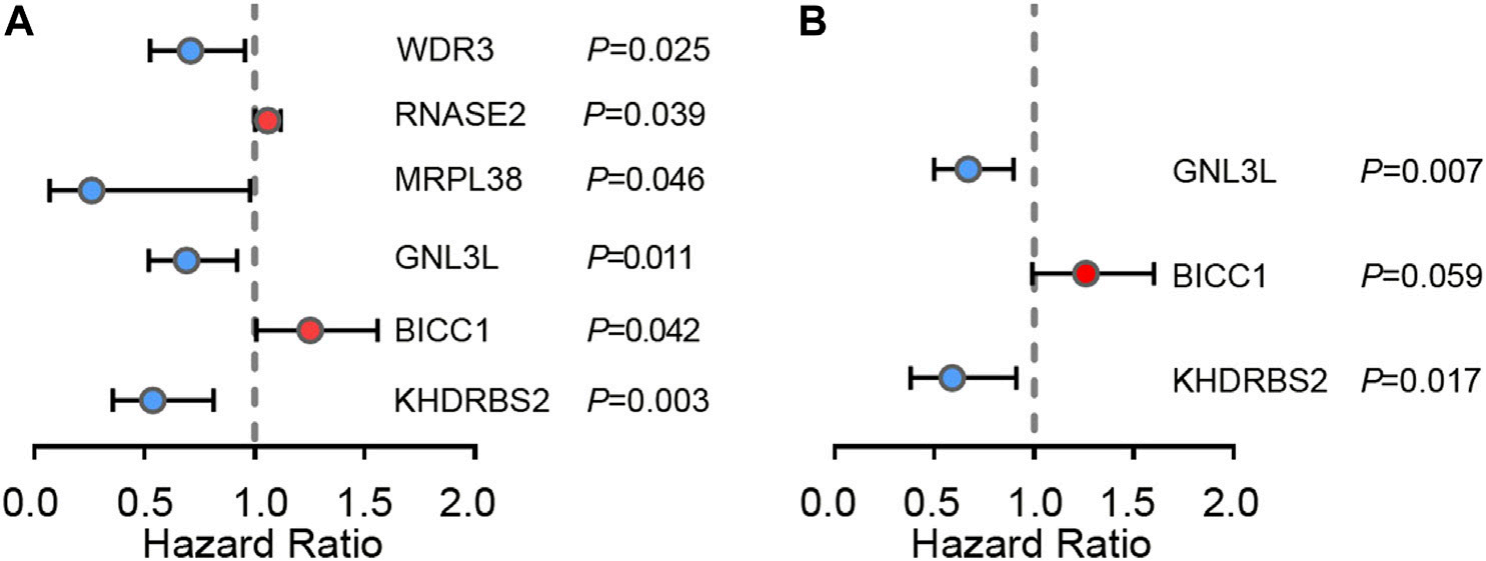
Cox regression analysis in TCGA GBM cohort. **(A)** Univariate Cox regression analysis for identification of hub RBPs in TCGA dataset. **(B)** Multivariate Cox regression analysis to identify prognosis related hub RBPs.

In order to study the pivotal DERBPs in GBM, we utilized Cytoscape software to create a PPI network, containing 120 nodes and 260 edges (Supplementary Figure S2). To deeply evaluate the prognosis values of 120 RBPs, univariate Cox regression analysis of each for OS was conducted and we obtained 6 prognosis-associated candidate hub RBPs. As shown in Figures 3A, 2 RBPs displayed an inverse correlation with survival, and 4 RBPs exhibited a positive correlation with survival (*p* < 0.05) (Figure 3A and Table 2). Following that, multiple stepwise Cox regression was used to evaluate the influence of the 6 potential hub RBPs on patient survival time and clinical outcomes. We eventually identified 3 hub RBPs, including G Protein Nucleolar 3 Like (GNL3L), BicC Family RNA Binding Protein 1 (BICC1), and KH RNA Binding Domain Containing, Signal Transduction Associated 2 (KHDRBS2), correlating with OS (Figure 3B and Table 2). High expression of BICC1 was found in GBM and its expression was inversely correlated with survival (HR > 1). Low expression of KHDRBS2 was found in GBM and its level was positively correlated with survival (HR < 1). Of interest, expression of GNL3L was dramatically higher in GBM, but its increase in expression exhibited a positive correlation with survival (HR < 1). Our results collectively implied that these 3 hub RBPs had close relations with the OS in GBM patients.

**TABLE 2:** 3 prognosis-associated hub RBPs identified by univariate and multivariate Cox regression analysis.

Factor	Up/Down	Univariate analysis			Multivariate analysis			
		HR	95% CI	*p* Value	β	HR	95% CI	*p* Value
BICC1	Up	1.253	1.007–1.558	0.042	0.231	1.259	0.990–1.602	0.059
RNASE2	Up	1.059	1.002–1.118	0.039				
GNL3L	Up	0.691	0.520–0.920	0.011	−0.527	0.669	0.498–0.898	0.007
KHDRBS2	Down	0.538	0.355–0.814	0.003	−0.402	0.590	0.382–0.911	0.017
WDR3	Down	0.709	0.525–0.957	0.025				
MRPL38	Down	0.260	0.069–0.979	0.046				

Abbreviations: HR, hazard ratio; CI, confidence interval.

### The Survival and Treatment Response was Predicted by the 3 Hub RBPs-Derived Risk Score Model in TCGA GBM Cohort

**FIGURE 4:**
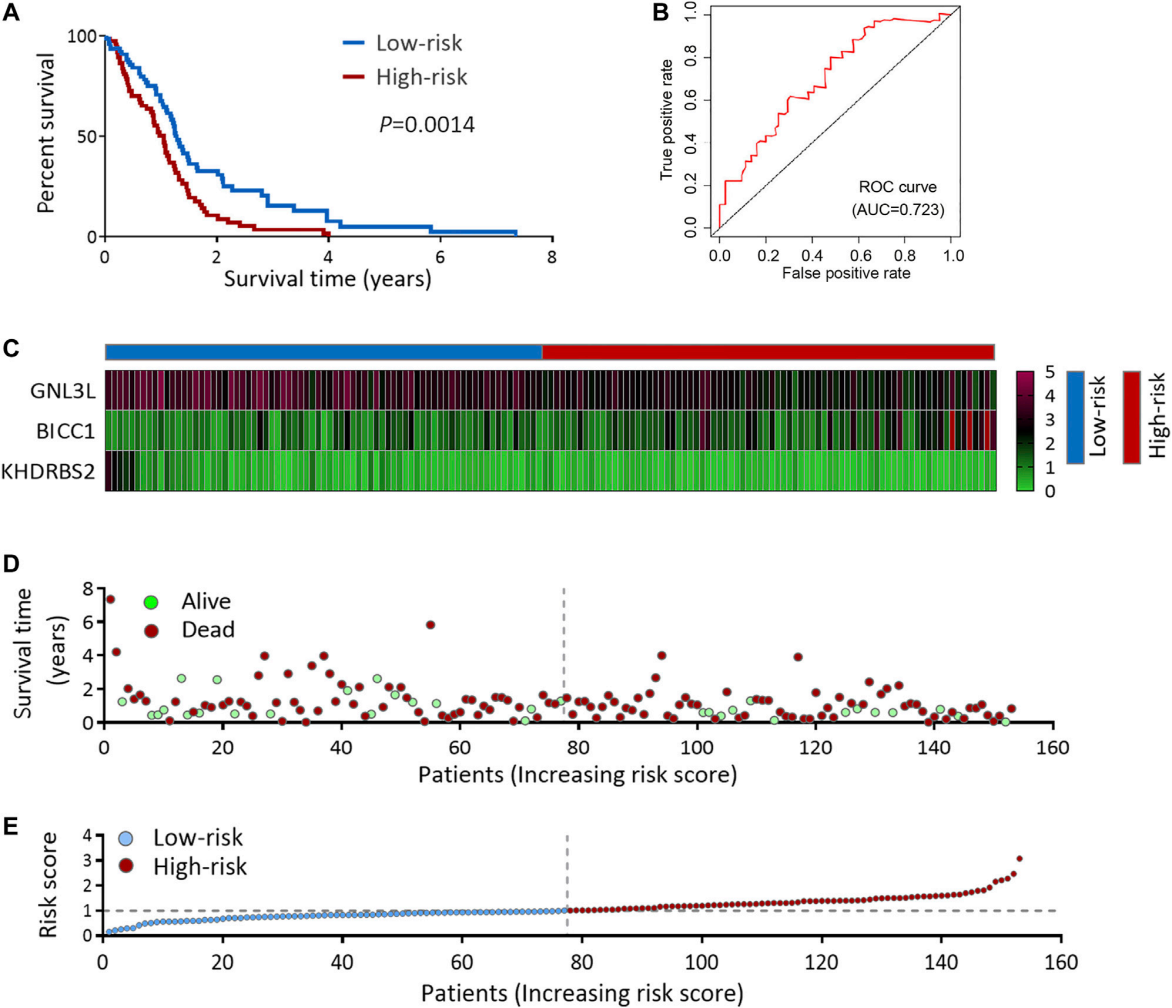
Risk score analysis of 3-genes prognostic model in TCGA cohort. **(A)** OS analysis among TCGA GBM patients stratified by risk score. **(B)** ROC curve for forecasting OS based on risk score. **(C)** Expression heat map of the 3 hub RBPs. **(D)** Survival status of the TCGA GBM patients. **(E)** The risk score values in low- and high-risk subgroups. OS, overall survival.

Next, a risk score model was built according to the above 3 hub RBPs. Each patient’s risk score got acquired from the formula below: $\text{Risk score}=\beta_{\text{BICC}1}{\text{×E}}_{\text{BICC}1}+\beta_{\text{GNL}3\text{L}}{\text{×E}}_{\text{GNL}3\text{L}}+\beta_{\text{KHDRBS}2}{\text{×E}}_{\text{KHDRBS}2}.$ “E” indicates the expression values of pertinent RBPs, and “*β*” represents the regression coefficient determined from multivariate Cox stepwise regression analysis according to the TCGA GBM cohort (Table 2). Then, we assessed predictive ability by conducting a survival analysis. TCGA GBM patients got classified into low- and high-risk subgroups with the standard of the median value. The data suggested compared with the low-risk group, the high-risk group presented an unsatisfying OS status (Figure 4A). A time-dependent ROC analysis was used to further assess the predictive capability of the 3 RBPs-derived risk model. Our data revealed RBPs risk score model’s area under the ROC curve (AUC) was 0.723 (Figure 4B), suggesting it displayed a moderate diagnosis ability. Figures 4C–E showed the expression heat maps of the 3 hub RBPs, patient survival status and the risk score of the signature comprised of 3 RBPs in the low and high-risk subgroups.

**FIGURE 5:**
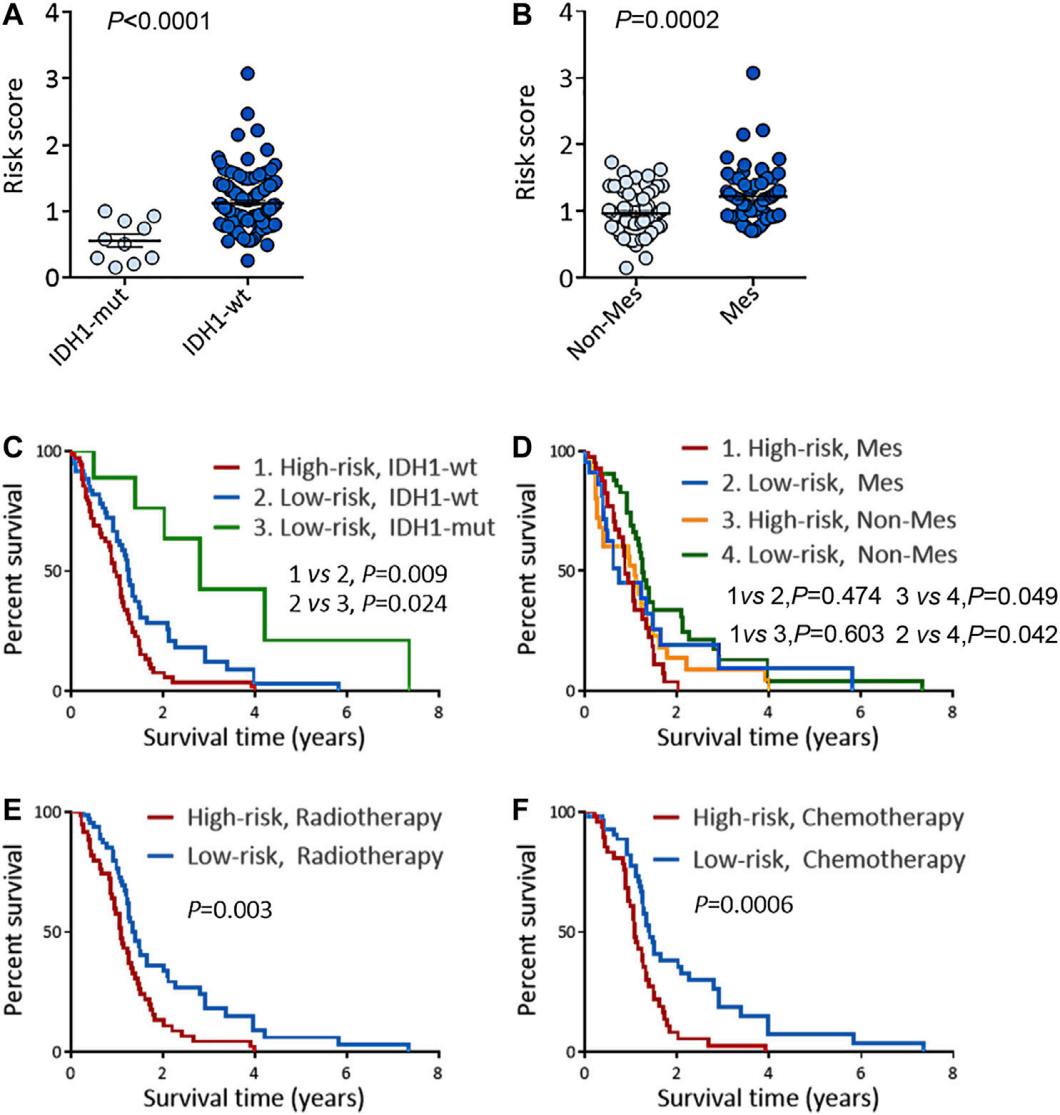
Risk score predicts the prognosis and treatment response in TCGA GBM cohort. **(A)** Risk scores in IDH1-mut GBM and IDH1-wt GBM. **(B)** Risk scores in Non-Mes GBM and Mes GBM. **(C–D)** KM OS analysis of TCGA GBM patients stratified by risk score combined with IDH1 status **(C)** and combined with expression subtypes **(D)**. **(E–F)** KM OS analysis of TCGA GBM patients with radiotherapy **(E)**, or TMZ chemotherapy **(F)** according to the risk score. KM, Kaplan-Meier; OS, overall survival; IDH, isocitrate dehydrogenase; IDH1-wt, IDH1-wild type; IDH1-mut, IDH1-mutation; Mes, mesenchymal; Non-Mes, non-mesenchymal; TMZ, temozolomide. Data are shown as mean ± SEM.

A high-risk score was strongly linked with IDH1-wild type and mesenchymal subtype, according to an analysis of the correlation between the risk score and clinicopathological features (Figures 5A,B and Supplementary Table S1). The IDH1-wild type or mesenchymal subtype GBM was linked to a bad prognosis (Supplementary Figure S3), as shown in earlier research (Yan et al., 2009; Wang et al., 2017), implying that a high-risk score might be associated with an unsatisfying prognosis.

KM survival analysis of the above two groups was performed to confirm the risk score’s prognosis relevance. Survival analysis indicated GBM patients with a high-risk score and IDH1-wild type had the worst results (Figure 5C), and in the non-mesenchymal subgroup, patients with a high-risk score had a shorter survival time than those with a low-risk score (Figure 5D). Univariate and multivariate analyses were employed to validate the prognosis significance of risk score (Supplementary Table S2). Moreover, in GBM patients suffering from radiation or TMZ chemotherapy, a high-risk score was linked to a poor result, showing the risk score might predict the treatment effects (Figures 5E,F).

### The Prognosis and Treatment Response was Predicted by the Risk Score Model in CGGA GBM Cohort

**FIGURE 6:**
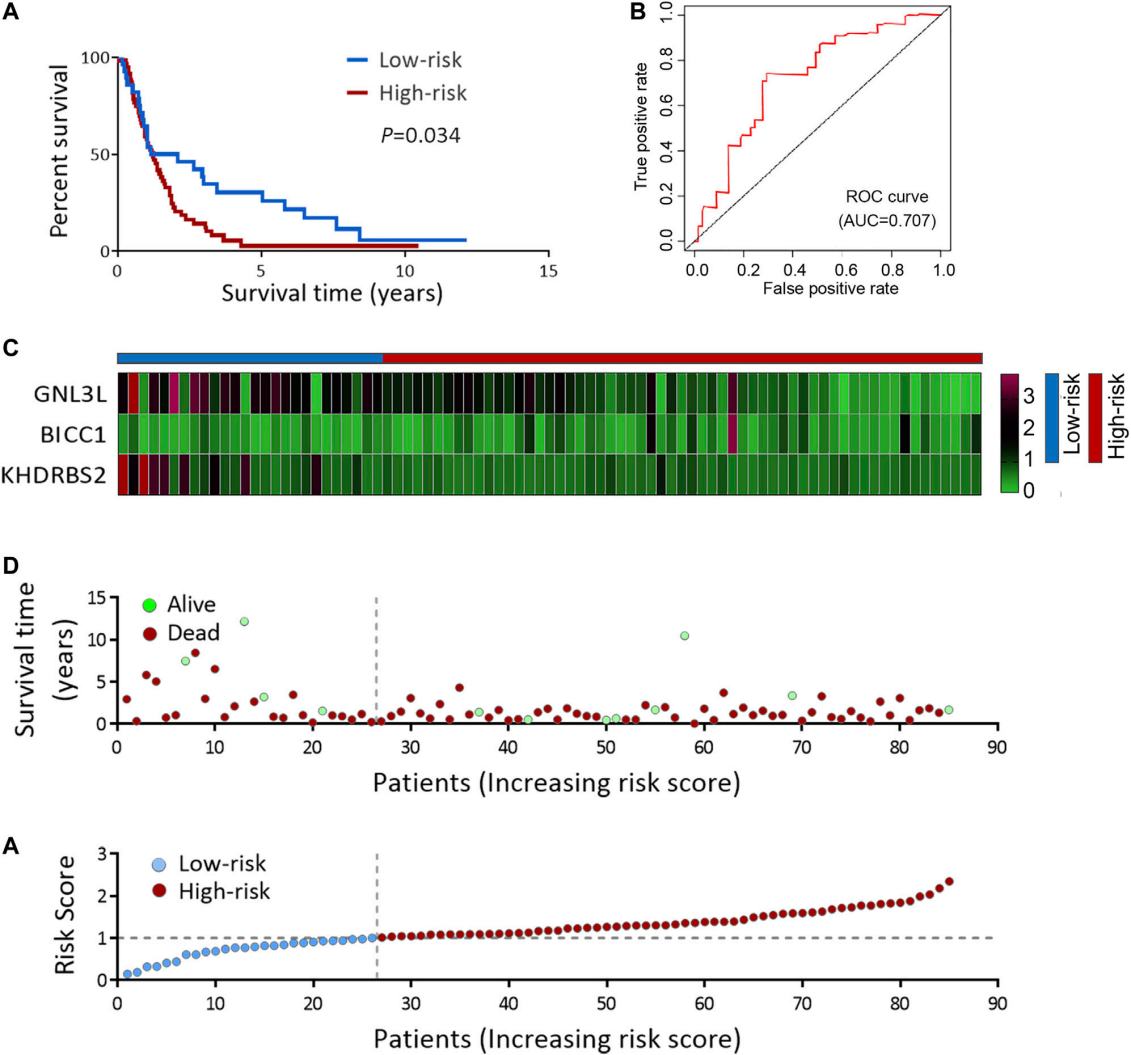
Risk score analysis of 3-genes prognostic model in CGGA cohort. **(A)** OS analysis among CGGA GBM patients stratified by risk score. **(B)** ROC curve for forecasting OS based on risk score. **(C)** Expression heat map of the 3 hub RBPs. **(D)** Survival status of the CGGA GBM patients. **(E)** The risk score values in low- and high-risk subgroups. OS, overall survival.

**FIGURE 7:**
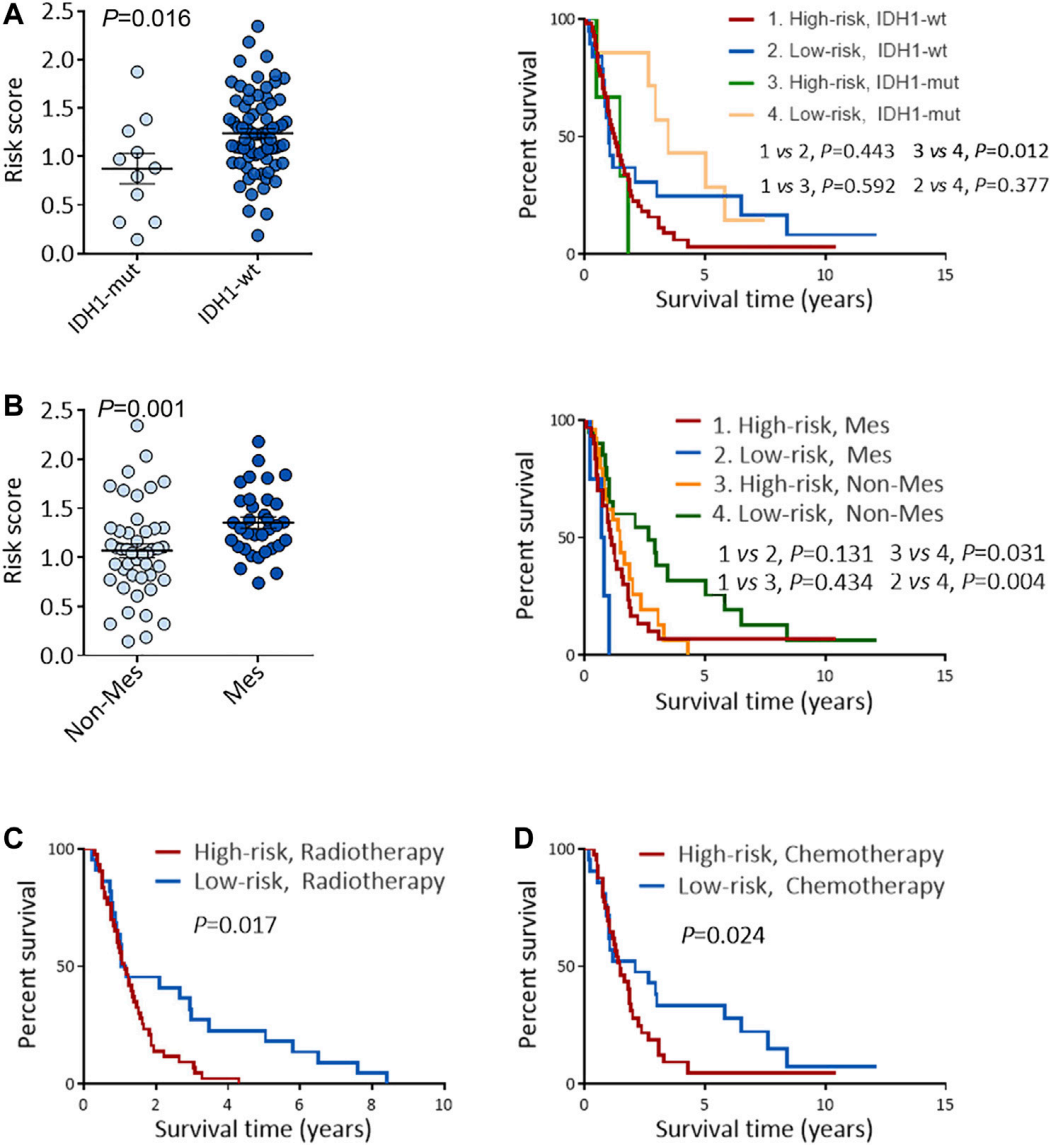
Performance of risk score in predicting the survival and treatment response in CGGA GBM cohort. **(A)** Risk scores in IDH1-mut GBM and IDH1-wt GBM (left), and KM OS analysis of CGGA GBM patients stratified by risk score combined with IDH1 status (right). **(B)** Risk scores in Non-Mes GBM and Mes GBM (left), KM OS analysis of CGGA GBM patients stratified by risk score combined with expression subtypes. **(C–D)** KM OS analysis of CGGA GBM patients with radiotherapy **(C)**, or TMZ chemotherapy **(D)** according to the risk score. KM, Kaplan-Meier; OS, overall survival; IDH, isocitrate dehydrogenase; IDH1-wt, IDH1-wild type; IDH1-mut, IDH1-mutation; Mes, mesenchymal; Non-Mes, non-mesenchymal; TMZ, temozolomide. Data are shown as mean ± SEM.

To deeply confirm the prediction ability of risk score, the 3 hub RBPs-derived risk score model was assessed, and KM survival analysis got performed in CGGA dataset of GBM. It was found that those with a high-risk score got a worse OS than patients with a low-risk score (Figure 6A). The AUC of the prognostic model counted 0.707, which showed a similar result compared to AUC in TCGA cohort (Figure 6B). Figures 6C–E showed the expression heat maps of 3 hub RBPs, patient survival status, and the risk score of the signature comprised of 3 RBPs in low- and high-risk subgroups. We found that compared to GBM patients with IDH1-mutation and a low-risk score, those with IDH1-mutation and a high-risk score tended to have a poor outcome, while no obvious survival difference was observed in GBM patients with a high-risk or low-risk score in the IDH1-wild type subgroup (Figure 7A), probably because of tumor heterogeneity and small sample size. Similarly, in comparison with non-mesenchymal GBM patients having low-risk scores, those having high-risk scores had worse survival time (Figure 7B). Finally, a high-risk score was linked with bad results in GBM patients undergoing radiotherapy or TMZ chemotherapy (Figures 7C,D). Collectively, these findings suggested the risk score model could forecast GBM patients’ prognosis and therapy evaluation.

### Nomogram Design on the Basis of the Hub RBPs

**FIGURE 8:**
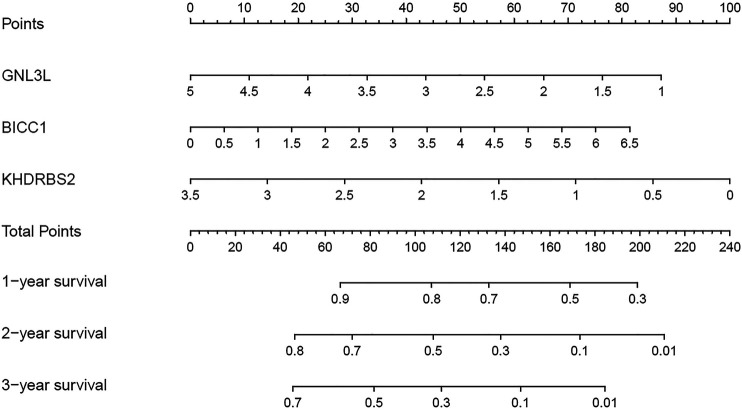
Nomogram for predicting 1, 2, and 3-years OS of GBM patients in the TCGA cohort.

3 RBPs signatures were combined for nomogram construction to create a predictive estimation approach (Figure 8). According to the multivariate Cox analysis, points were distributed to respective variables through the nomogram scale. A horizontal line was drawn for each variable with demarcations for the number of points, the whole points were counted for each patient and generalized to a range of 0–100. The evaluated survival rates of GBM patients could be determined by drawing a vertical line from the total point axis to each prognosis axis in the next 3 years. This nomogram would facilitate the clinical treatment for GBM patients.

### Validating the Differentially Expressed 3 Hub RBPs in GBM Cells

**FIGURE 9:**
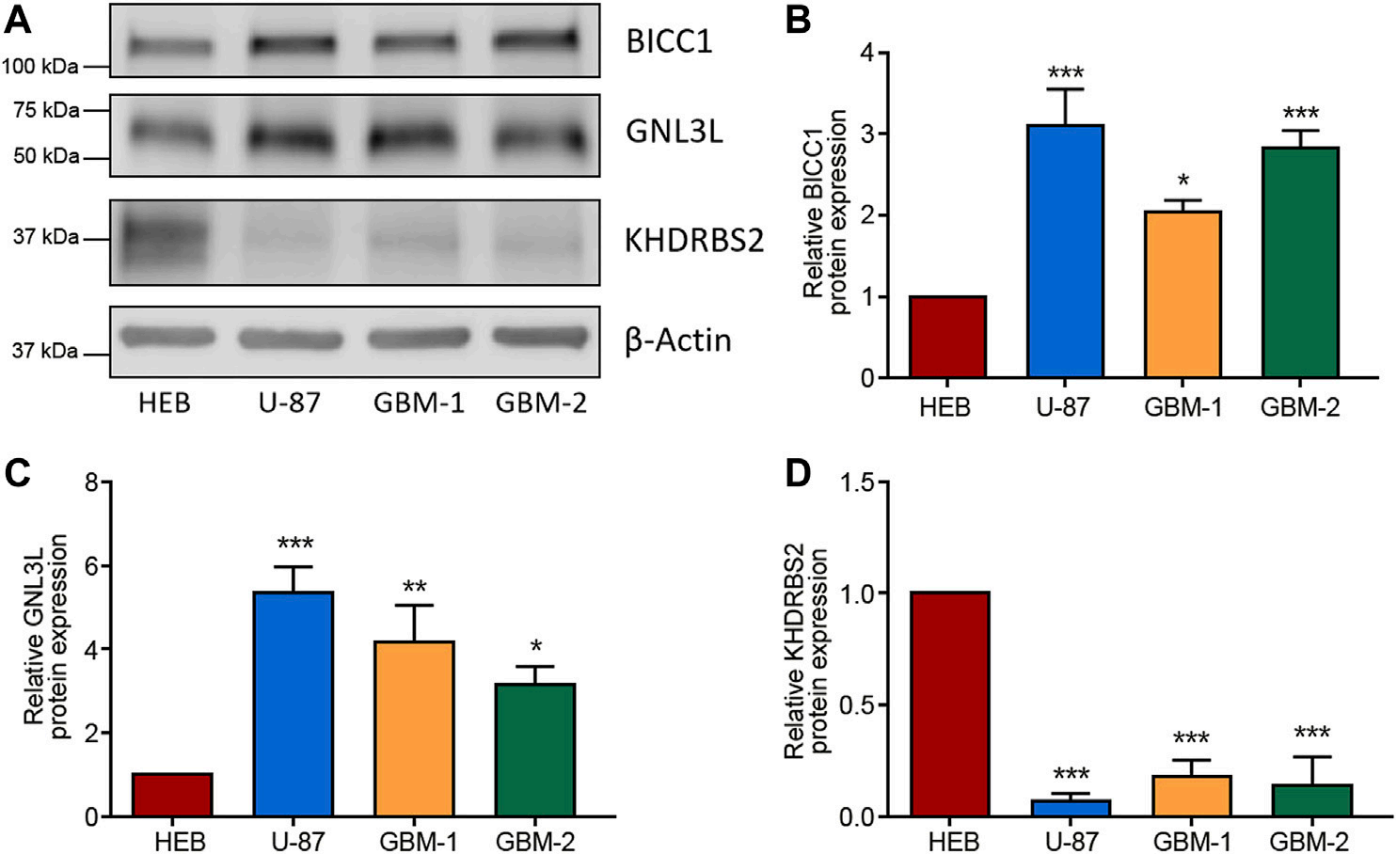
Validation of the differential expression of the 3 RBPs in GBM cells. **(A–D)** Western blot images **(A)** and the relevant quantification of BICC1 **(B)**, GNL3L **(C)**, and KHDRBS2 **(D)** in GBM cell line U-87, primary GBM cells (GBM-1 and GBM-2), and normal glial cell line HEB. The relative expression of target proteins is quantified in comparison with β-Actin and normalized to the corresponding expression in HEB cells. Data are shown as mean ± SEM from five independent experiments, *p < 0.05, **p < 0.01, ***p < 0.001.

To confirm the dysregulated expressions of the 3 hub RBPs (BICC1, GNL3L, and KHDRBS2) in GBM, western blot was carried out to measure their protein levels in GBM cells and normal glial cells. Figure 9 indicated BICC1 and GNL3L were upregulated in GBM cells, while KHDRBS2 was downregulated.

## Discussion

GBM is featured with complicated background on the molecular level (Reifenberger et al., 2016), while high-throughput data on GBM studies makes it easier to find the molecular diagnosis and prognosis biomarkers. In our context, we comprehensively investigated the DERBPs of GBM from TCGA and GTEx cohorts, and uncovered 3 hub RBPs (BICC1, GNL3L, and KHDRBS2) abnormally expressing in GBM. The risk score model originated from the 3 hub RBPs exhibited vital functions in the prediction of GBM patients’ prognosis and treatment response.

Firstly, we screened for the GBM DERBPs through RNA sequencing data from TCGA and GTEx datasets, rather than only TCGA. The normal brain tissue samples in TCGA were acquired from tissues surrounding tumors, which couldn’t represent normal brain tissue samples completely. The GTEx dataset contained 1,092 samples of 11 brain regions from 201 normal individuals (Consortium, 2015; Mu et al., 2019). In this way, the discovered RBPs (52 upregulated and 108 downregulated) might surely have a stable and particular expression in GBM than that in normal control. We built co-expression and PPI networks for these RBPs by thoroughly studying key biological pathways. Next, we discovered that BICC1, GNL3L, and KHDRBS2 levels were associated with survival by univariate and multivariate Cox regression analysis. Among them, upregulated BICC1 exhibited a negative correlation with survival, indicating it functioned as an oncogene. Downregulated KHDRBS2 displayed a positive correlation with survival, so it might function as a tumor suppressor gene in GBM. Of note, expression of GNL3L was considerably higher in GBM while being positively correlated with survival, so GNL3L was an indicator of lower risk in GBM (HR < 1). In addition, western blot analysis confirmed that the 3 hub RBPs were differentially expressed in GBM cells. As a result, the risk score model was created using the signature of the 3 hub RBPs.

According to prior research, regulation of translation, RNA processing and RNA metabolism are all linked to the incidence and progression of a range of human diseases (Subramanian and Simon, 2010; Caffarel and Coleman, 2014; Reifenberger et al., 2016). In our study, the functional enrichment analysis indicated the abnormal RBPs governed the carcinogenesis and development of GBM *via* the mRNA surveillance pathway, RNA degradation, ribosome synthesis, and RNA degradation. Although most connections between RBPs and tumors remain confusing, there are still some findings of them (Wang et al., 2018). For instance, PTBP1 can enhance glioma proliferation and migration by increasing the inclusion of exon 3 in RTN4 mRNA (Cheung et al., 2009). HNRNPA2B1 promotes glioma development and aggressiveness (Golan-Gerstl et al., 2011). PTRF, alias Cavin1, is recognized as a non-canonical RBP in GBM and is also identified as a prognosis-related factor (Huang et al., 2018; Wang et al., 2020a). In addition, FNDC3B, a membrane protein, not only promotes migration and invasion of glioma cells, but can also act as a prognostic biomarker (Fischer et al., 2017; Wang et al., 2020a). SLC25A43, a molecular marker, is also proved to be related to a poor prognosis in GBM (Haitina et al., 2006; Wang et al., 2020a).

Herein, the 3 hub RBPs-derived risk score performed well in predicting the GBM patients’ survival status in TCGA, and their prognosis functions could be reproduced in CGGA. According to the ROC curve study, the 3 RBPs signatures had the diagnosis ability to determine the GBM patients with a bad prognosis. The constructed nomogram facilitated the OS prediction in the following 3 years more quantitatively. In addition, the risk score model could forecast the treatment effects of GBM patients undergoing radiotherapy or chemotherapy. Therefore, a prospective risk score model derived from multi-RBPs signature was established, which could be utilized as a biomarker for GBM’s prognosis and prediction.

Among the 3 hub RBPs, BICC1 is implicated in the post-transcriptional regulation of mRNA (Rothé et al., 2015; Davidson et al., 2016). Inhibition of BICC1 expression can promote cell apoptosis and suppress cell proliferation in tumor cells (Wang et al., 2020b). GNL3L, HSR1-MMR1 family, is a putative nucleolar GTPase existing throughout eukaryotes (Thoompumkal et al., 2016). GNL3L has been discovered as a factor involved in the maintenance of the tumorigenic properties of tumor-initiating cells (Kannathasan et al., 2020). Moreover, GNL3L may enhance NF-κB-regulated tumor cell viability via the upregulation of antiapoptosis-related genes (Thoompumkal et al., 2015; Kannathasan et al., 2020). However, no previous study has assessed the effects of GNL3L in GBM. As for KHDRBS2, although the relation of KHDRBS2 overexpression to better OS in lung adenocarcinoma is well understood (Li et al., 2020b), little is known about it in GBM. The biological activities of these 3 hub RBPs have offered some insight into the value of risk score in GBM prognosis and prediction, but more research of them in GBM development and the potential mechanisms is needed.

The risk score’s applicable efficacy may be beneficial for directing treatment options to improve the clinical outcome of GBM patients. Patients with high-risk scores should accept aggressive treatment, while those with low-risk scores should avoid excessive therapies that might result in unwanted side effects. As a result, it is critical to put the risk score into clinical practice that is guaranteed by promising research to deeply confirm the value of risk score in GBM prediction and prognosis.

This study has some limitations. First, the number of clinical samples used to verify 3 hub RBPs is not large enough. Secondly, the biological functions of these 3 RBPs in GBM need to be further explored. Finally, the clinical application value of risk scores remains to be further verified.

Our systematic exploration of DERBPs through a sequence of bioinformatics analyses in GBM, and identified a total of 160 DERBPs (52 upregulated and 108 downregulated). The results of functional analysis showed that RBPs are mainly involved in mRNA surveillance pathway, RNA degradation, ribosome synthesis, and RNA degradation. Univariate and multiple COX regression analysis showed that BICC1, GNL3L, and KHDRBS2 are related to the prognosis of GBM patients. A risk score model was constructed based on the differential expressions of 3 hub RBPs. In addition, we analyzed the expression levels of 3 hub RBPs in GBM tissues and cell lines. This risk score model performs favorably in the prediction of GBM patients’ therapy and prognosis, which potentially optimizes treatment decisions.

## Data Availability

The original contributions presented in the study are included in the article/Supplementary Material, further inquiries can be directed to the corresponding authors.
