# Supplementary figures and images for: Development of a 3 RNA Binding Protein Signature for Predicting Prognosis and Treatment Response for Glioblastoma Multiforme
Source: Front Genet. 2021 Oct 18;12:768930. doi: 10.3389/fgene.2021.768930 (PMC8558313; doi:10.3389/fgene.2021.768930)

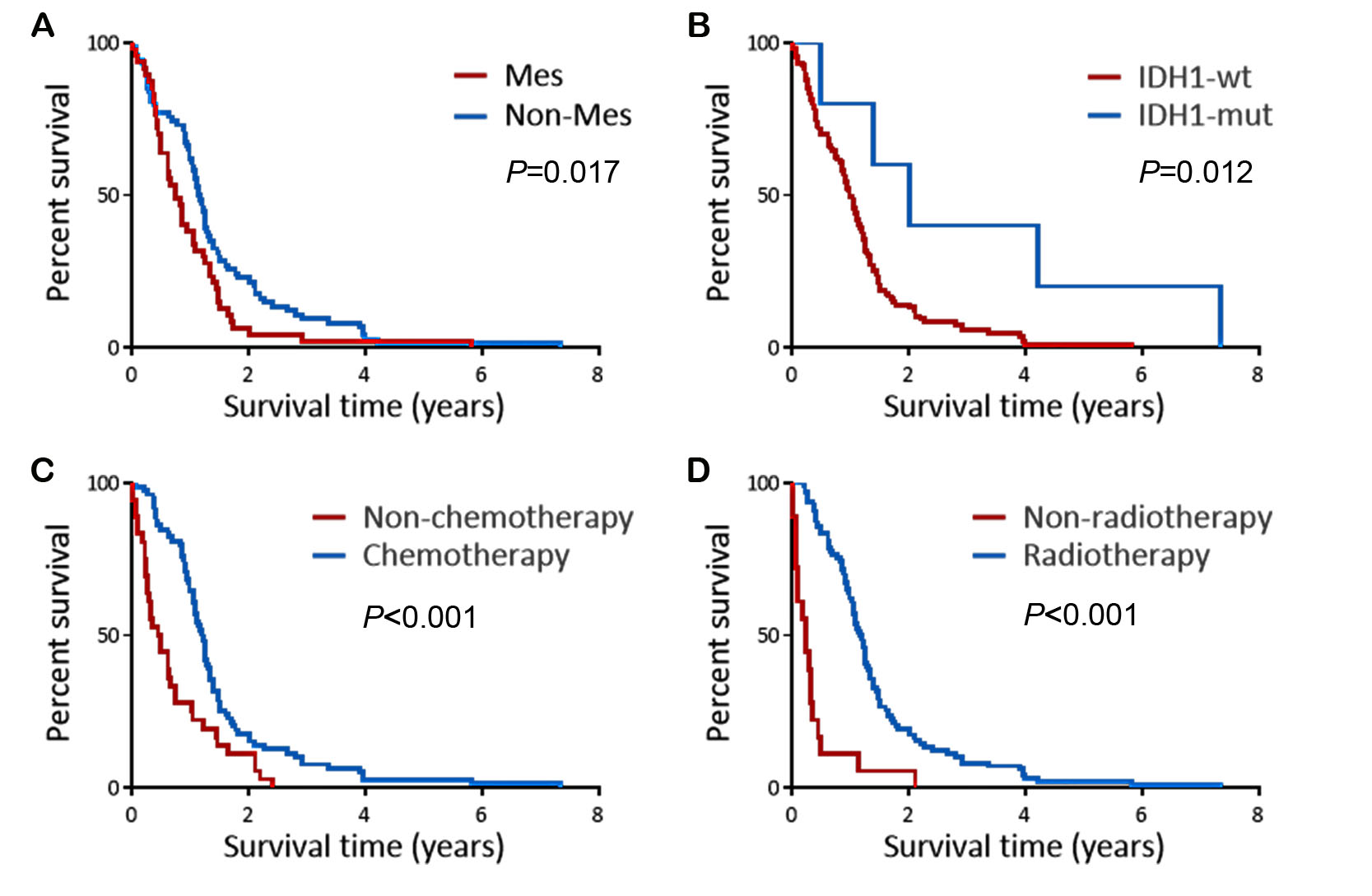

Supplement: Supplementary file 1 [file Image3.JPEG]

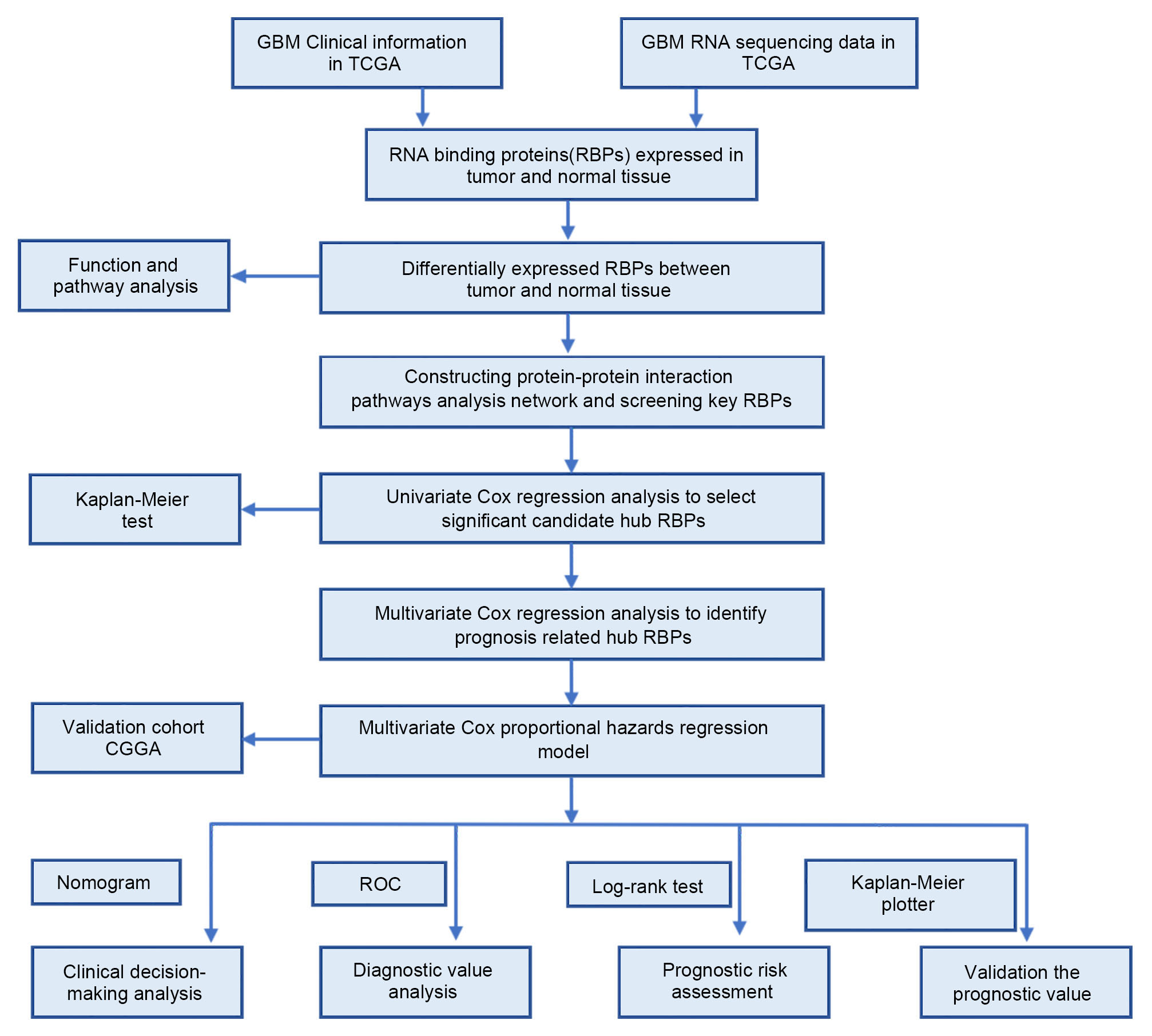

Supplement: Supplementary file 3 [file Image1.JPEG]
