# Supplementary material for: Development of a 3 RNA Binding Protein Signature for Predicting Prognosis and Treatment Response for Glioblastoma Multiforme
Source: Front Genet. 2021 Oct 18;12:768930. doi: 10.3389/fgene.2021.768930 (PMC8558313; doi:10.3389/fgene.2021.768930)
Supplement: Supplementary file 2 [file Table1.docx]

| **Table S1** The clinical characteristics of GBM patients in TCGA cohort. | | | | | | |  |
| --- | --- | --- | --- | --- | --- | --- | --- |
| **Characteristic** | |  | **Risk score** | | |  | ***P* value** |
|  |  |  | Low (*n*=77) | | High (*n*=76) | |  |
| Gender |  |  |  |  |  |  | 0.241 |
|  | Male |  | 48 (62.34%) | | 48 (63.16%) | |  |
|  | Female |  | 25 (32.47%) | | 24 (31.58%) | |  |
|  | NA |  | 4 (5.19%) | | 4 (5.26%) | |  |
| Age |  |  |  |  |  |  | 0.549 |
|  | >60 |  | 34 (44.16%） | | 38 (50.00%) | |  |
|  | ≤60 |  | 39 (50.65%） | | 34 (44.74%) | |  |
|  | NA |  | 4 (5.19%) | | 4 (5.26%) | |  |
| Expression subtypes | | |  |  |  |  | 0.003**** |
|  | Mes |  | 22 (28.57%) | | 26 (34.21%) | |  |
|  | No-Mes |  | 42 (54.55%) | | 40 (52.63%) | |  |
|  | NA |  | 13 (16.88%) | | 10 (16.16%) | |  |
| IDH1 status | |  |  |  |  |  | 0.002**** |
|  | Mutation |  | 10 (12.99%) | | 0 (0%) |  |  |
|  | Wild type | | 61 (79.22%) | | 70 (92.11%) | |  |
|  | NA |  | 6 (7.79%) | | 6 (7.89%) | |  |
| MGMT status | |  |  |  |  |  | 0.003**** |
|  | Methylated | | 28 (36.36%) | | 19 (25.00%) | |  |
|  | Unmethylated | | 29 (37.66%) | | 36 (47.37%) | |  |
|  | NA |  | 20 (25.97%) | | 21 (27.63%) | |  |
| Radiotherapy | |  |  |  |  |  | 0.295 |
|  | Yes |  | 67 (87.01%) | | 61 (80.26%) | |  |
|  | No |  | 8 (10.39%) | | 11 (14.47%) | |  |
|  | Missing |  | 2 (2.60%) | | 4 (5.26%) | |  |
| Chemotherapy | |  |  |  |  |  | 0.4435 |
|  | Yes |  | 55 (71.43%) | | 49 (64.47%) | |  |
|  | No |  | 19 (24.68%) | | 21 (27.63%) | |  |
|  | NA |  | 3 (3.90%) | | 6 (7.89%) | |  |
|  | | | | | | | |
| **Abbreviations:** IDH1, isocitrate dehydrogenase 1; MGMT, O(6)-methylguanine-DNA methyltransferase. *** P* < 0.01*.* | | | | | | | |
