# Supplementary material for: Development of a 3 RNA Binding Protein Signature for Predicting Prognosis and Treatment Response for Glioblastoma Multiforme
Source: Front Genet. 2021 Oct 18;12:768930. doi: 10.3389/fgene.2021.768930 (PMC8558313; doi:10.3389/fgene.2021.768930)
Supplement: Supplementary file 5 [file Table2.docx]

| **Table S2** Univariate and multivariate Cox regression analysis of clinicopathological parameters and risk score for overall survival in TCGA | | | | | | | | | | | | | | | | | | | | | | | |  |
| --- | --- | --- | --- | --- | --- | --- | --- | --- | --- | --- | --- | --- | --- | --- | --- | --- | --- | --- | --- | --- | --- | --- | --- | --- |
| GBM cohort. | | | | | | | | | | | | | | | |  |  |  | | | |  |  |  |
| **Factor** | **Univariate analysis** | | | | | | | |  | |  | | **Multivariate analysis** | | | | | | | | | | |  |
|  | HR (95%CI) | | | | *P* value | | | |  | |  |  | HR (95%CI) | | | | | | | *P* value | | | |  |
| Age | 1.030 (1.015, 1.047) | | |  | | < 0.001 | | | |  | |  | | | 1.016 (0.996, 1.035) | | | |  | | 0.125 | | | |
| Gender | 0.906 (0.610, 1.347) | | |  | | 0.626 | | | |  | |  | | | 1.197 (0.769, 1.862) | | | |  | | 0.426 | | | |
| Expression subtypes | 1.466 (0.986, 2.181) | | |  | | 0.059 | | | |  | |  | | | 1.193 (0.775, 1.836) | | | |  | | 0.422 | | | |
| IDH1 status | 0.237 (0.095, 0.591) | | |  | | 0.002 | | | |  | |  | | | 0.667 (0.215, 2.071) | | | |  | | 0.484 | | | |
| MGMT status | 0.446 (0.274, 0.724) | | |  | | 0.001 | | | |  | |  | | | 0.574 (0.326, 1.009) | | | |  | | 0.054 | | | |
| Radiotherapy | 0.172 (0.102, 0.290) | | |  | | < 0.001 | | | |  | |  | | | 0.188 (0.088, 0.400) | | | |  | | < 0.001 | | | |
| Chemotherapy | 0.372 (0.247, 0.558) | | |  | | < 0.001 | | | |  | |  | | | 0.429 (0.263, 0.700) | | | |  | | 0.001 | | | |
| Risk score | 2.589 (1.724, 3.887) | | |  | | < 0.001 | | | |  | |  | | | 2.338 (1.495, 3.656) | | | |  | | < 0.001 | | | |
|  | |  |  | | | |  |  | | | | | |  | |  |  |  | | | |  |  |  |
| **Abbreviations**: IDH1, isocitrate dehydrogenase 1; MGMT, O(6)-methylguanine-DNA methyltransferase; HR, hazard ratio; CI, confidence interval. | | | | | | | | | | | | | | | | | | | | | | | |  |
|  | |  |  | | | |  |  | | | | | |  | |  |  |  | | | |  |  |  |
